# Clinical efficacy of traditional Chinese medicine compound on patients with knee osteoarthritis and its impact on inflammatory cytokine levels

**DOI:** 10.12669/pjms.41.7.10724

**Published:** 2025-07

**Authors:** Kui Wang, Zuoyuan Wu, Xiangnan Shen, Yanjun Wang, Chenglin Mu

**Affiliations:** 1 Kui Wang, Department of Orthopedics and Traumatology, Hebei Province Hospital of Chinese Medicine, Shijiazhuang 050000, Hebei, China; 2 Zuoyuan Wu, Department of Orthopedics and Traumatology, Hebei Province Hospital of Chinese Medicine, Shijiazhuang 050000, Hebei, China; 3 Xiangnan Shen, Department of Orthopedics and Traumatology, Hebei Province Hospital of Chinese Medicine, Shijiazhuang 050000, Hebei, China; 4 Yanjun Wang, Department of Acupuncture, Hebei Province Hospital of Chinese Medicine, Shijiazhuang 050000, Hebei, China; 5 Chenglin Mu, Department of Orthopedics and Traumatology, Hebei Province Hospital of Chinese Medicine, Shijiazhuang 050000, Hebei, China

**Keywords:** Clinical efficacy, Inflammatory factors, Knee osteoarthritis, Medicine compound, Traditional Chinese

## Abstract

**Objective:**

To investigate the clinical efficacy of traditional Chinese medicine (TCM) compound in the treatment of knee osteoarthritis (KOA) and its impact on inflammatory cytokine levels.

**Methods:**

A retrospective analysis was conducted on the medical records of 80 KOA patients admitted to Hebei Province Hospital of Chinese Medicine from January 2021 to January 2023. The patients were divided into an observation group and a control group based on different treatment methods (n=40 each group). The visual analogue scale (VAS) was adopted to assess patients’ pain level, and the Lysholm score was used to evaluate knee joint function, and the clinical outcomes of the two groups were compared.

**Results:**

After treatment, both groups exhibited a significant decrease in VAS scores, with the observation group showing a more substantial reduction(P<0.05). The Lysholm scores of both groups were significantly higher after treatment than before treatment (P<0.05). Serum levels of CRP, IL-6, and TNF-α decreased significantly in both groups after treatment, with the observation group showing a more pronounced reduction (P<0.05). The ADL scores for both advanced and basic abilities improved significantly post-treatment in both groups, with the observation group demonstrating a statistically significant greater improvement (P<0.05). The treatment efficacy for the observation group was 92.50%, which was significantly higher than the control group’s rate of 77.50% (χ²=4.114, P<0.05).

**Conclusion:**

Traditional Chinese Medicine(TCM) compound therapy for KOA can significantly alleviate patients’ pain, reduce the expression of inflammatory factors in serum, enhance knee joint function, and improve patients’ quality of life.

## INTRODUCTION

Osteoarthritis (OA) is a chronic orthopedic condition characterized by progressive articular cartilage degeneration, joint destruction, and hyperostosis^1^,^2^, which can affect multiple joints of the human body. This disease predominantly affects the knee joint, making it the most susceptible to OA.^3^ Knee osteoarthritis (KOA), also known as degenerative osteoarthritis of the knee joint, is mainly caused by age-related degenerative alterations and chronic strain of the knee joint, leading to cartilage degeneration and osteophyte development. It is notably prevalent among the middle-aged and senior demographics, with evidence indicating that up to 60% of individuals over the age of 55 may suffer from KOA, with a higher incidence observed in females compared to males.^4^

Clinically, KOA is characterized by symptoms such as joint swelling and pain. As the condition advances, patients may experience increased knee stiffness and atrophy of the adjacent musculature, which can severely impair joint mobility and emerge as a leading contributor to lower limb functional limitations in the elderly.^5^ The escalating prevalence of KOA, exacerbated by the aging population in China, underscores its significance as a major public health concern. Traditional Chinese Medicine(TCM) categorizes KOA within the “bi syndrome” and “crane knee wind” classifications, attributing its etiology to a combination of liver and kidney insufficiency and the pathological influence of wind, cold, and dampness.

The primary TCM syndrome differentiation centers on liver and kidney deficiency, presenting with wind-cold-dampness arthralgia. The foundational therapeutic approaches in TCM aim to nourish qi and blood, dispel wind, eliminate dampness, warm the meridians, disperse cold, and enhance blood circulation to alleviate stasis.^6^ Conventional clinical management of KOA typically involves nonsteroidal anti-inflammatory drugs (NSAIDs), intra-articular injections, and surgical interventions such as total joint arthroplasty. Despite their effectiveness, these treatments present challenges, particularly for the middle-aged and elderly populations. NSAIDs are associated with potential gastrointestinal and cardiovascular side effects, intra-articular injections carry a risk of infection, and surgical procedures are invasive and costly.^7^,^8^

Consequently, there is a pressing need for a treatment method that is safe, low-risk, efficacious, and widely acceptable. In this context, TCM therapies have demonstrated promising outcomes in the management of KOA. This study investigated the clinical efficacy of a specific TCM compound administered orally and topically in treating KOA. It further examines the compound’s influence on inflammatory cytokine levels, thereby contributing valuable insights into the potential of TCM-based interventions for KOA management.

## METHODS

A retrospective analysis was conducted on the medical records of 80 KOA patients admitted to Hebei Province Hospital of Chinese Medicine from January 2021 to January 2023. The patients were divided into an observation group and a control group based on different treatment methods, with 40 patients in each group.

### Ethics approval:

The study was approved by the Institutional Ethics Committee of Hebei Province Hospital of Chinese Medicine (No.: 2023-123; Date: December 1, 2023), and written informed consent was obtained from all participants.

### Inclusion criteria:

- A confirmed diagnosis of KOA according to the criteria set forth in the “Chinese Guidelines for the Diagnosis and Treatment of Osteoarthritis (2021 Edition)” and “TCM Guidelines for Knee Osteoarthritis (2020 Edition)”.
- A Kellgren-Lawrence classification of grades I to III as determined by radiographic assessment.
- Aged 55-75 years old, with complete clinical data.
- No use of KOA-related treatment drugs for at least one month before treatment.
- No known allergy to topical application of TCM formulations.
- Informed consent of patients along with a commitment to comply with the full course of the treatment regimen.

### Exclusion criteria:

- The presence of cutaneous lesions or disruptions in the vicinity of the knee joint.
- A medical history indicative of serious comorbid conditions.
- A history of gastrointestinal ulcers.
- TCM allergy constitution.

The control group received conventional treatment, including oral administration of anti-inflammatory and analgesic medication, nutritional supplements for articular cartilage, topical application of proprietary Chinese medicines, and intra-articular injection of sodium hyaluronate, with continuous treatment for five weeks. The observation group was given additional treatment with a compound traditional Chinese medicine (TCM) formula for both oral consumption and topical application on top of the conventional treatment. The orally administered TCM compound included: 12 g each of Herba Lycopodii, Himalayan Coralbean, Mulberry Twig, and Common Achyranthes; 10 g each of Cowherb Seed, Safflower, Camphor Wood, Clematis chinensis, Tuberculate Speranskia Herb, and Sappan Wood; decocted in water, one dose per day, taken twice daily in the morning and evening. The method for the TCM compound topical application: the basic composition of the topical formula included 10 g each of Chinese Angelica, Papaya, Manchurian Wildginger Herb, Prepared Common Monkshood Mother Root, Safflower, Earthworm, Prepared Kusnezoff Monkshood Mother Root, Achyranthis Bidentatae Radix, and Pine Nodular Branch. The decoction was first prepared and then poured into a medicinal pad, which was then soaked and tightly applied to both sides of the knee joints, fixed in place with a bandage. The topical application was done once every night for eight hours. Patients in both groups received continuous treatment for five weeks, and followed up for five months.

### Observation Indicators:

- Before treatment and five weeks after treatment, pain assessment was conducted using the Visual Analogue Scale (VAS), with scores ranging from 0 (no pain) to 10 (severe pain). Knee joint function was evaluated using the Lysholm scoring system, which has a total score of 0 to 100; higher scores indicate better knee joint function.
- Three ml of fasting blood samples from the median cubital vein of patients were collected before and after treatment to measure levels of C-reactive protein (CRP), Interleukin-6 (IL-6), and tumor necrosis factor-alpha (TNF-α).
- Activities of Daily Living (ADL): The patients’ ability to perform daily activities was assessed using the Activity of Daily Living Scale before and after treatment, with a total score ranging from 0 to 100. Higher scores suggest a stronger ability for self-care.
- Clinical efficacy was evaluated based on the TCM syndrome scale in the “TCM Guidelines for the Diagnosis and Treatment of Knee Osteoarthritis (2021 Edition)”: cured: TCM clinical symptoms and signs basically disappeared after treatment; markedly effective: TCM clinical symptoms and signs significantly improved after treatment; effective: TCM clinical symptoms and signs mildly improved after treatment; ineffective: no relief or even aggravation of TCM clinical symptoms and signs after treatment. The effective rate is calculated as the sum of the cure rate, markedly effective rate, and effective rate.

### Statistical analysis:

SPSS 21.0 software was used for analysis. Quantitative data were expressed as mean ± standard deviation (*χ̅*±*S*), and the t-test was used for inter-group comparison. Qualitative data were expressed as the number of cases and percentage (%), and the χ^2^ test was used for inter-group comparison. P<0.05 was considered statistically significant.

## RESULTS

There were no statistically significant differences in age, duration of illness, gender, or Kellgren-Lawrence grading of knee osteoarthritis on X-ray imaging (P>0.05) between the two groups, indicating comparability between the groups, Table-I.

**Table-I:** Comparison of general information between the two groups.

Group	n	Age (years)	Duration of illness (months)	Gender (male/female)	K-L grading		
					Grade-I	Grade-II	Grade-III
Observation group	40	64.75±2.87	16.15±1.03	21/19	20(50.00)	13(32.50)	7(17.50)
Control group	40	65.33±2.87	16.10±1.53	20/20	18(45.00)	18(45.00)	4(10.00)
*t/x^2^*		0.896	0.171	0.050	1.730		
*P*		0.373	0.864	0.823	0.421		

Before treatment, there was no statistically significant difference in VAS and Lysholm scores between the two groups (P>0.05). After treatment, the VAS scores of both groups decreased significantly, with a greater reduction in the observation group compared to the control group (P<0.05). The Lysholm scores of both groups were significantly higher after treatment than before treatment (P<0.05), with a more significant increase in the observation group compared to the control group (P<0.05), Table-II.

**Table-II:** Comparison of VAS and Lysholm scores between the two groups before and after treatment (*χ̄*±*S*).

Group	VAS score		Lysholm score	
	Before treatment	After treatment	Before treatment	After treatment
Observation group	7.80±0.72	1.90±0.59	35.93±2.80	83.53±4.09
Control group	7.85±0.70	2.35±0.62	35.23±2.99	77.90±3.71
*t*	0.314	3.318	1.081	6.447
*P*	0.754	0.001	0.283	0.000

Before treatment, there was no statistically significant difference in the levels of CRP, IL-6, and TNF-α between the two groups (P>0.05). After treatment, the levels of CRP, IL-6, and TNF-α in both groups were significantly lower than before treatment, with a more significant decrease in the observation group (P<0.05), Table-III.

**Table-III:** Comparison of inflammatory indicators between the two groups before and after treatment (*χ̄*±*S*).

Observation indicator	Observation point	Observation group	Control group	t	p
CRP(mg/L)	Before treatment	37.17±3.69	35.92±3.49	1.558	0.123
	After treatment	13.51±2.50	18.66±3.34	7.813	0.000
IL-6 (ng/L)	Before treatment	112.48±7.64	112.20±6.09	0.183	0.855
	After treatment	53.74±6.11	57.67±5.52	3.025	0.003
TNF-α(ng/L)	Before treatment	1.75±0.18	1.73±0.16	0.567	0.573
	After treatment	1.10±0.10	1.28±0.07	9.186	0.000

Before treatment, there was no statistically significant difference in ADL advanced ability and basic ability scores between the two groups (P>0.05). After treatment, the ADL advanced ability and basic ability scores of both groups improved significantly compared to before treatment, with a statistically significant difference between the groups (P<0.05) and a greater improvement in the observation group (P<0.05), Table-IV. After treatment, the effective rate of the observation group was 92.50%, significantly higher than 77.50% in the control group (P<0.05), Table-V.

**Table-IV:** Comparison of ADL scores between the two groups before and after treatment (*χ̄*±*S*).

Group	n	Advanced ability		Basic ability	
		Before treatment	After treatment	Before treatment	After treatment
Observation group	40	5.38±0.98	23.83±2.68	19.53±1.63	43.65±2.44
Control group	40	5.33±1.02	18.73±1.75	19.10±1.43	39.33±1.91
*t*		0.223	10.075	1.239	8.811
*P*		0.824	＜0.001	0.219	＜0.001

**Table-V:** Comparison of clinical efficacy between the two groups [n,(%)].

Group	Cured	Markedly effective	Effective	Ineffective	Total effective rate
Observation group	9(22.50)	20(50.00)	9(22.50)	2(5.00)	38(95.00)
Control group	8(20.00)	16(40.00)	8(20.00)	8(20.00)	32(80.00)
*χ²*					4.114
*P*					0.043

## DISCUSSION

The results of this study show that compared with before treatment, the levels of CRP, IL-6, and TNF-α in both groups significantly decreased after treatment (P<0.05), with a more substantial reduction in the observation group compared to the control group (P<0.05). This suggests that the combination of oral administration and topical application of TCM compound can significantly reduce the inflammatory response. We believe that oral administration of TCM can reduce the inflammatory response, while topical application can promote the penetration of effective ingredients of TCM into the joint tissues, which is conducive to the absorption of inflammatory substances, delaying the degeneration of articular cartilage and promoting the healing of the body.^9^-^11^ The pathological process of KOA involves various inflammatory cells, which can damage the normal structure of the cartilage matrix, leading to chondrocyte apoptosis, cartilage inflammation, and synovitis.^12^,^13^

KOA is a type of osteoarthritis associated with knee joint degeneration, degradation, and long-term wear, based on articular cartilage degeneration, degradation, and subchondral hyperostosis. In the late stage, joint effusion, deformity, or intra-articular loose bodies may occur. In advanced stages, joint effusion, deformities, or loose bodies within the joint may occur. Knee Osteoarthritis (KOA) is a significant disease that causes knee pain and mobility issues in middle-aged and elderly individuals.^14^,^15^ Current Western medical treatments for KOA include pharmacological and surgical interventions. Commonly used medications comprise oral Nonsteroidal Anti-Inflammatory Drugs (NSAIDs) and glucosamine, as well as intra-articular injections of hyaluronic acid. Oral NSAIDs can provide temporary pain relief; however, symptoms often recur after discontinuation of the drugs. Glucosamine can nourish joint cartilage and improve clinical symptoms but is less effective in reducing inflammation. Hyaluronic acid can lubricate the joint; however, like surgery, it is an invasive procedure with a risk of infection.^16^,^17^

Traditional Chinese Medicine (TCM) categorizes KOA under “bi syndrome,” where liver and kidney deficiencies and insufficient Qi and blood in middle age lead to the loss of nourishment in tendons and vessels. This, combined with the invasion of pathogenic factors such as wind, cold, and dampness into the joints, obstructs the flow of Qi and blood. Overexertion, falls, or injuries can also cause blood stasis in the meridians, affecting the circulation of Qi and blood. Treatment in TCM primarily focuses on nourishing Qi and blood, strengthening the kidneys, and fortifying tendons, as well as dispelling wind and eliminating dampness. The internal and external remedies used in this study mainly aim to remove dampness, relieve pain, relax tendons, invigorate collaterals, dispel wind, and tonify blood.^18^,^19^

The primary clinical manifestations of KOA are knee joint pain and mobility impairment due to pain. The condition is often prolonged and progressively worsens, causing serious negative impacts on patients’ physical health, quality of life, and economic situation.^20^ The purpose of treatment is to alleviate joint pain and improve patients’ quality of life.^21^ The results of this study show that after treatment, the efficiency of patients in the observation group was 92.50%, higher than 77.50% in the control group (χ²=4.114,P<0.05), and the VAS score was significantly lower than that of the control group (P<0.05), while the Lysholm and ADL scores were significantly higher (P<0.05). The results indicate that the combination of oral administration and topical application of TCM compound in the treatment of KOA has a definite therapeutic effect. This treatment method can not only quickly alleviate patients’ joint pain and improve joint function but also significantly enhance patients’ quality of life, achieving the purpose of treating both the symptoms and the root cause.

### Limitations:

However, this study also has some shortcomings, such as small sample size, subjective influence on knee joint function assessment, and the possibility of patients’ own functional exercise affecting the efficacy, further in-depth research with larger samples and objective evaluation indicators is needed in the future.

## CONCLUSIONS

TCM compound treatment for KOA has a significant therapeutic effect, which may alleviate patients’ pain symptoms, improve knee joint function, and enhance patients’ quality of life.

### Authors’ Contributions:

**KW** and **CM:** Performed the studies, participated in collecting data, drafted the manuscript, and are responsible and accountable for the accuracy or integrity of the work. **ZW, XS** and **YW:** Literature search, Performed the statistical analysis and participated in its design. All authors have read and approved the final manuscript.
